# Practice Guidelines on NAFLD

**DOI:** 10.1002/hep.25998

**Published:** 2013-01-04

**Authors:** Melissa Palmer

**Affiliations:** New York UniversityDix Hills, NY

To The Editor:

I read with interest the practice guidelines on nonalcoholic fatty liver disease (NAFLD),^1^ in which the authors fail to reference the association between high fructose corn syrup (HFCS) and NAFLD. The intake of HFCS, a combination of glucose and fructose, has increased over time and parallels both the obesity and NAFLD epidemics.^2^ Numerous studies demonstrate that the mechanism by which HFCS causes NAFLD is due to fructose. In animal models, fructose causes steatosis and fibrosis^3^,^4^ by either increasing hepatic lipogenesis, causing activation of pyruvate dehydrogenase,^5^ activating inflammatory pathways,^6^ or up-regulating the expression of sterol regulatory element-binding protein (SREBP).^7^

In humans, excessive fructose intake can lead to increased hepatic lipid deposition, greater insulin resistance, and hypertriglyceridemia.^8^ NAFLD patients have been found to drink more HFCS soft drinks compared with healthy controls.^9^ A retrospective analysis of 341 NAFLD adults found that those consuming high fructose diets had more fibrosis than those consuming low fructose diets.^10^ A prospective controlled trial with histologic endpoints is needed to define the amount of HFCS safe for NAFLD patients and to determine the extent to which fructose contributes to the pathogenesis and progression of NAFLD. However, there are currently sufficient data to recommend that NAFLD patients refrain from excessive consumption of HFCS.
